# Long-term outcome after surgical ventricular septal defect closure: Longitudinal follow-up into the fifth decade

**DOI:** 10.1016/j.ijcchd.2025.100624

**Published:** 2025-10-10

**Authors:** Sahra Ünlütürk, Robert M. Kauling, Judith A.A.E. Cuypers, Annemien E. van den Bosch, Alexander Hirsch, Chiara Pelosi, Daniel J. Bowen, Raluca G. Chelu, Ad J.J.C. Bogers, Willem A. Helbing, Isabella Kardys, Jolien W. Roos-Hesselink

**Affiliations:** a Department of Cardiology, Cardiovascular Institute, Thoraxcenter, Erasmus MC, Rotterdam, the Netherlands; b European Reference Network for Rare, Low Prevalence and Complex Diseases of the Heart (ERN GUARD-Heart), Amsterdam, the Netherlands; c Department of Radiology and Nuclear Medicine, Erasmus MC, Rotterdam, the Netherlands; d Department of Cardiothoracic Surgery, Thorax Center, Erasmus MC, Rotterdam, the Netherlands; e Department of Pediatrics, Division of Cardiology, Erasmus MC, University Medical Center Rotterdam, Rotterdam, the Netherlands; f Clinical Epidemiology and Innovation Unit, Department of Cardiology, Erasmus MC, University Medical Center Rotterdam, Rotterdam, the Netherlands

**Keywords:** Ventricular septal defect, Surgical VSD closure, Cardiac surgery, Long-term outcome, Quality of life

## Abstract

**Objectives:**

To evaluate survival, clinical outcome and quality of life (QoL) of patients up to 49 years after surgical ventricular septal defect (VSD) closure.

**Methods:**

Single-center, longitudinal cohort study evaluating consecutive patients with surgical VSD closure between 1968 and 1980 with extensive cardiac and QoL evaluation every decade.

**Results:**

Of the original cohort of 174 patients, 39 died (22 %), 8 were lost to follow-up and 38 had not participated previously. Survival rate at 49 years follow-up was 77 % and 86 % when excluding early postoperative mortality. Of the 89 eligible survivors, 76 (85 %) were evaluated (59 % male, median age 49 [44–54] years) with a median follow-up of 44 (range 40–49) years after surgery. Event-free survival at 49 years was 50 %, with symptomatic arrhythmias (10 %), pacemaker implantation (8 %) and VSD-related interventions (3 %) being common complications. At last follow-up, 58 % had left atrial dilation, 25 % had aortic regurgitation and 5 patients (7 %) had a residual VSD. Early postoperative arrhythmias predicted mortality. Both left ventricular (LV) and right ventricular ejection fraction remained stable, with only 1 % having an LV ejection fraction below 45 % at last follow-up. Exercise capacity and VO_2_max were mildly reduced in 33 % and 49 % of the patients while self-perceived QoL was stable and comparable with the general Dutch population.

**Conclusion:**

Half of the patients with surgical VSD closure had an event-free survival at 49 years. Pacemaker implantation was often needed. Early postoperative arrhythmias predicted mortality. QoL was good and remained stable over time.

## Graphical abstract

This central illustration summarizes the long-term follow-up of patients who underwent surgical ventricular septal defect (VSD) closure during childhood. Survival at 49 years was 77 %. Event-free survival was 50 %, with arrhythmias (10 %), pacemaker implantation (8 %), and VSD-related interventions (3 %) as the most frequent complications. PR and QRS prolongation, left atrial dilation and aortic regurgitation were common. Early postoperative arrhythmias predicted mortality. Despite these findings, ventricular function was largely preserved and exercise capacity only mildly reduced. Importantly, self-reported quality of life was stable and comparable to the general population, highlighting favorable long-term functional outcomes in this aging cohort.

**Figure:**
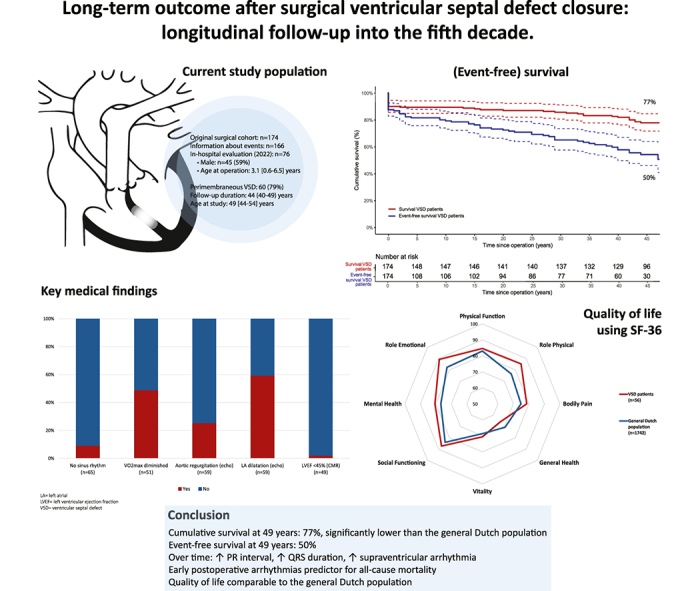

## 1 Introduction

Ventricular septal defect (VSD) is the most prevalent congenital heart defect (CHD), with a prevalence of 3 per 1000 live-births [1]. VSDs often occur in isolation, but may be associated with or be part of other CHDs, such as aortic coarctation or Tetralogy of Fallot. The size of the defect, along with the ratio of pulmonary/systemic vascular resistance, determine the shunt volume and direction of blood flow. Small defects may not cause significant hemodynamic changes, whereas a large shunt can lead to left ventricular overload and dysfunction, as well as the development of pulmonary arterial hypertension, which may result in reduced life expectancy, heart failure or arrhythmias. In subaortic VSDs, aortic regurgitation may also occur [2]. Surgical closure has long been the only treatment, and the long-term outcomes are generally considered favorable [3]. As a result, most patients are discharged from routine cardiological follow-up [4]. However, it is crucial for both patients and healthcare providers to understand whether the preoperative left-to-right shunt and the surgical VSD closure have long-term effects on life-expectancy, morbidity and quality of life (QoL). Studies reporting on the long-term outcome after surgical VSD closure during childhood are scarce.

The present analysis describes the fourth investigation of a cohort of consecutive patients who underwent surgical VSD closure at young age between 1968 and 1980. The aim is to address the gaps in knowledge by evaluating the long-term health outcomes of this cohort, assessing whether there are ongoing cardiovascular or functional concerns that were not evident in their earlier years. By systematically following this group, we seek to shed light on potential challenges they may face, which could improve future care strategies for similar patients.

## 2 Methods

### 2.1 Study population

This study is a single-center longitudinal study evaluating consecutive patients with VSD who underwent surgical VSD closure in the Erasmus Medical Center between 1968 and 1980 at young age (<15 years), with follow-up every decade. This cohort was previously described in 1990, 2001 and 2012 [3,5,6]. In this analysis, patients with concomitant lesions were grouped as ‘’nonisolated VSD’’. First, survival was checked for the total cohort. All surviving patients who had participated in at least one of the prior investigations were invited for this new in-hospital evaluation. Alongside detailed cardiological assessments, QoL evaluation was conducted using the Short-Form-36 (SF-36) questionnaire. For patients unable to attend the in-hospital evaluation, consent was obtained to use data from their medical records and a questionnaire was sent to retrieve up-to-date information. The study protocol was approved by the local Medical Ethics Committee (MEC 2019-0465) and written informed consent was obtained from all study participants. The study was carried out in accordance with to the principles of the Declaration of Helsinki.

### 2.2 Outcome

Survival status of the entire cohort was obtained from the Dutch National Population Registry (BRP) and compared to the age-matched general Dutch population (GDP). Adverse events were defined as all-cause mortality, cardiac reinterventions (both surgical and catheter-based), symptomatic arrhythmia, pacemaker implantation, implantable cardioverter-defibrillator (ICD) implantation, heart failure, stroke and endocarditis. Reintervention referred to all procedures related to the closure of a residual VSD performed after the primary surgery, while additional interventions included all other procedures. Symptomatic arrhythmias were defined as the prescription of anti-arrhythmic medication, hospitalization for arrhythmia, cardioversion or an ablation procedures. Heart failure was considered clinically relevant when a patient was prescribed heart failure medication or required hospitalization for heart failure.

### 2.3 In-hospital evaluation

Cardiac evaluation comprised 12-lead surface electrocardiography (ECG), 24-h ambulatory Holter monitoring, cardiopulmonary exercise testing (CPET), transthoracic echocardiography, cardiovascular magnetic resonance (CMR) and laboratory measurements (Supplementary Table 1). All echocardiographic studies were performed and analysed following the current guidelines [[7], [8], [9], [10]]. Systolic left and right ventricular function (LVF and RVF) were both measured and graded qualitatively. Elevated pulmonary pressure was defined as a tricuspid regurgitant jet velocity >2.8 m/s in the absence of pulmonary stenosis or right ventricular (RV) outflow tract obstruction, or as a pulmonary regurgitant jet velocity >2.2 m/s. CMR results, aligned with current reference values, were presented [11]. Patients' subjective health status was assessed using the SF-36 questionnaire with results compared to those of the age-matched GDP [12].

### 2.4 Statistical analysis

A detailed description of the statistical analysis is provided in Supplementary File 1.

## 3 Results

### 3.1 Study patients

**Fig. 1:**
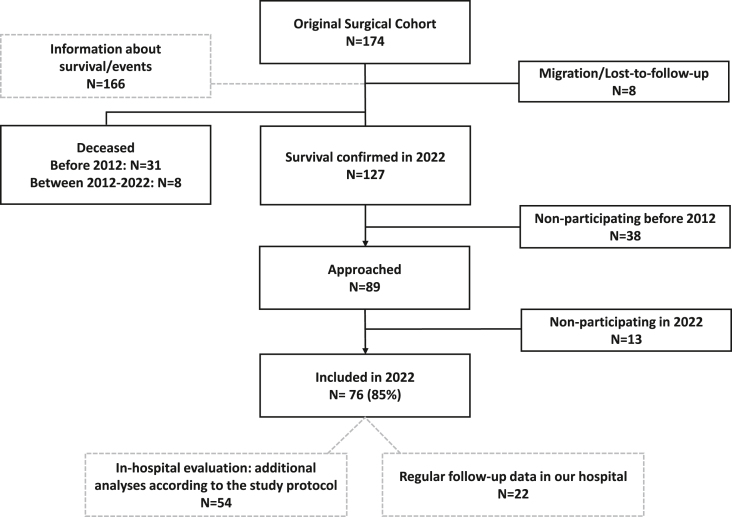
Current study participation This flowchart presents the inclusion of VSD patients who previously underwent surgical VSD closure. From the original surgical cohort of 174 patients, survival data were available for 166 patients. A total of 39 patients had died, and 8 patients had either migrated or were lost to follow-up. In 2022, 54 patients participated in comprehensive in-hospital evaluation, while data from an additional 22 patients were retrieved from routine clinical follow-up at our center. The remaining patients did not participate in the most recent in-hospital evaluation.

Current patient inclusion is visualized in Fig. 1. The original study cohort consisted of 174 consecutive patients (55 % male, age at operation 2.3 [0.5–6.9] years). Most patients had an isolated VSD (68 %). In 71 % of the patients, a Dacron patch was used during surgical VSD closure. Seventy-six of the eligible 89 patients (85 %) were included for the in-hospital evaluation in the present study (2022). Baseline characteristics are presented in Table 1 and Supplementary Table 2B. Median age at operation and at the time of the present study was 3.1 [0.6–6.5] and 49 [44–54] years respectively. Median follow-up was 44 (range 40–49) years after surgery. There were no differences in baseline characteristics between participating and nonparticipating patients (Supplementary Table 2A).

**Table 1:** Baseline characteristics.

	Total	1990	2001	2012	2022
	(n = 174)	(n = 107)	(n = 95)	(n = 91)	(n = 76)
Male	96 (55 %)	64 (60 %)	57 (60 %)	56 (62 %)	45 (59 %)
Age at operation, years	2.3 [0.5–6.9]	3.4 [0.6–7.1]	2.1 [0.6–6.5]	2.9 [0.5–6.5]	3.1 [0.6–6.5]
Age at operation <1 year	60 (34 %)	34 (32 %)	33 (35 %)	30 (33 %)	24 (32 %)
Pre-operative RV systolic pressure, mmHg	70 [40–83]	69 [42–85]	68 [42–82]	68 [42–82]	68 [42–80]
Pre-operative Qp/Qs ratio	2.0 [1.7–2.8]	2.0 [1.7–2.8]	2.1 [1.7–2.9]	2.0 [1.7–2.8]	2.0 [1.7–2.8]
Type of VSD					
Perimembranous	134 (77 %)	91 (85 %)	78 (82 %)	74 (81 %)	60 (79 %)
Muscular	8 (5 %)	1 (1 %)	2 (2 %)	2 (2 %)	5 (7 %)
Nonisolated VSD^a^	56 (32 %)	32 (30 %)	29 (31 %)	27 (30 %)	19 (25 %)
Patent foramen ovale/atrial septal defect	25 (45 %)	17 (53 %)	7 (24 %)	15 (56 %)	11 (58 %)
Patent ductus arteriosus	13 (23 %)	7 (22 %)	7 (24 %)	6 (22 %)	4 (21 %)
Coarctation aortae	8 (14 %)	5 (16 %)	5 (17 %)	5 (19 %)	4 (21 %)
Pulmonary stenosis	15 (27 %)	13 (41 %)	10 (34 %)	10 (37 %)	7 (37 %)
Mitral stenosis	2 (4 %)	2 (6 %)	2 (7 %)	2 (7 %)	1 (5 %)
Other	8 (14 %)	3 (9 %)	3 (10 %)	2 (7 %)	1 (5 %)
Previous PA banding	15 (9 %)	6 (6 %)	4 (4 %)	6 (7 %)	4 (5 %)
Hypothermia					
Temperature <20 °C	70 (40 %)	38 (35 %)	37 (39 %)	33 (36 %)	28 (37 %)
Temperature 20–35 °C	101 (58 %)	67 (63 %)	56 (59 %)	56 (62 %)	47 (62 %)
Temperature unknown	3 (2 %)	2 (2 %)	2 (2 %)	2 (2 %)	1 (1 %)
RV incision	82 (47 %)	56 (52 %)	46 (48 %)	46 (51 %)	39 (51 %)
VSD closure with patch	155 (89 %)	95 (89 %)	86 (91 %)	80 (88 %)	64 (84 %)
Post-operative arrhythmia <30 days^b^	22 (13 %)	7 (7 %)	7 (7 %)	7 (8 %)	2 (3 %)
Heart block <30 days^b^	9 (5 %)	2 (2 %)	3 (3 %)	3 (3 %)	2 (3 %)
Follow-up, years	–	14 [[12], [13], [14], [15], [16]]	25 [[23], [24], [25], [26], [27]]	35 [30–37]	44 [43–46]
Age at study, years	–	19 [[14], [15], [16], [17], [18], [19], [20], [21], [22], [23], [24]]	29 [25–34]	40 [35–44]	49 [44–54]

a 14 patients had more than one concomitant lesion besides ventricular septal defect.

b *Some patients had ≥1* type of arrhythmia.

### 3.2 Survival

**Fig. 2:**
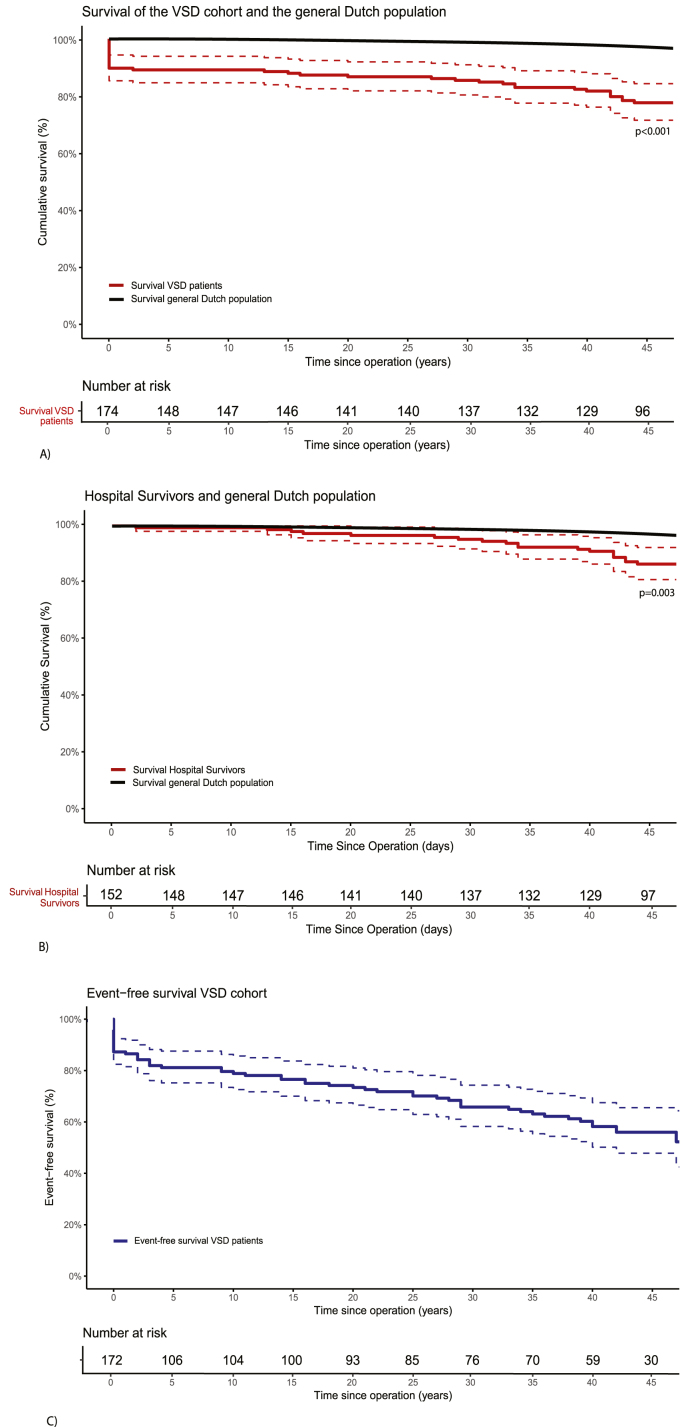
Survival of the VSD cohort and hospital survivors compared to the general Dutch population; event-free survival of the VSD cohort A) Survival plot describing survival in the studied cohort (at 49 years: 77 %, red) compared to the Dutch reference population (94.4 %, black) B) Survival plot describing survival in the hospital survivors (at 49 years: 86 %, red) compared to the Dutch reference population (94.4 %, black) C) Event-free survival of the VSD cohort (at 49 years: 50 %, blue) Panel A shows the cumulative survival (red) and event-free survival (blue) of the VSD cohort, compared to age- and sex-matched survival of the general Dutch population (black). After nearly 5 decades of follow-up, cumulative survival was 77 %, significantly lower than the general population (p < 0.001). Panel B shows the cumulative survival of hospital survivors (red), compared to age- and sex-matched survival of the general Dutch population (black). After 49 years of follow-up, survival among hospital survivors was 86 %, which remained significantly lower than the general population (94.4 %, p = 0.003). Panel C displays the event-free survival of the VSD cohort (blue). At 49 years of follow-up, 50 % of patients remained event-free.

Survival status was obtained for 166 of 174 patients (95 %). Eight patients either moved abroad or were untraceable. Cumulative survival of the entire cohort after surgical correction was 77 % at 49 years (Fig. 2A), and 86 % excluding in-hospital mortality (Fig. 2B). Both were significantly lower than the GDP survival of 94.4 % at 49 years (p < 0.001 and p = 0.003, respectively). Cumulative survival of patients with nonisolated and isolated VSD was 71 % and 80 % respectively (Supplementary Fig. 1A, p = 0.10). No significant difference in survival was observed between males and females (Supplementary Fig. 2A, p = 0.30). In total, 39 of the 174 patients died, of which 16 within 30 days after surgery. The incident mortality rate was 1.7 per 100 patient-years.

In the last 10 years, 8 patients died. One patient died due to stroke, 1 experienced sudden cardiac death (SCD), 1 died of COVID pneumonia and in 5 the cause was unknown. During the total follow-up, 3 patients suffered a SCD, all of whom had a RV incision during surgery. In Supplementary Table 3 an overview of all causes of death is provided.

### 3.3 Other major cardiac events

Cumulative event-free survival at 49 years was 50 % (Fig. 2C). Event-free survival for patients with nonisolated vs. isolated VSD was 24 % and 62 % respectively (Supplementary Fig. 1B, p = 0.001) with additional intervention being the most frequently occurring event. Event-free survival was similar between males and females (Supplementary Fig. 2B, p = 0.90). An overview of all recorded events is provided in Supplementary Table 4.

**Fig. 3:**
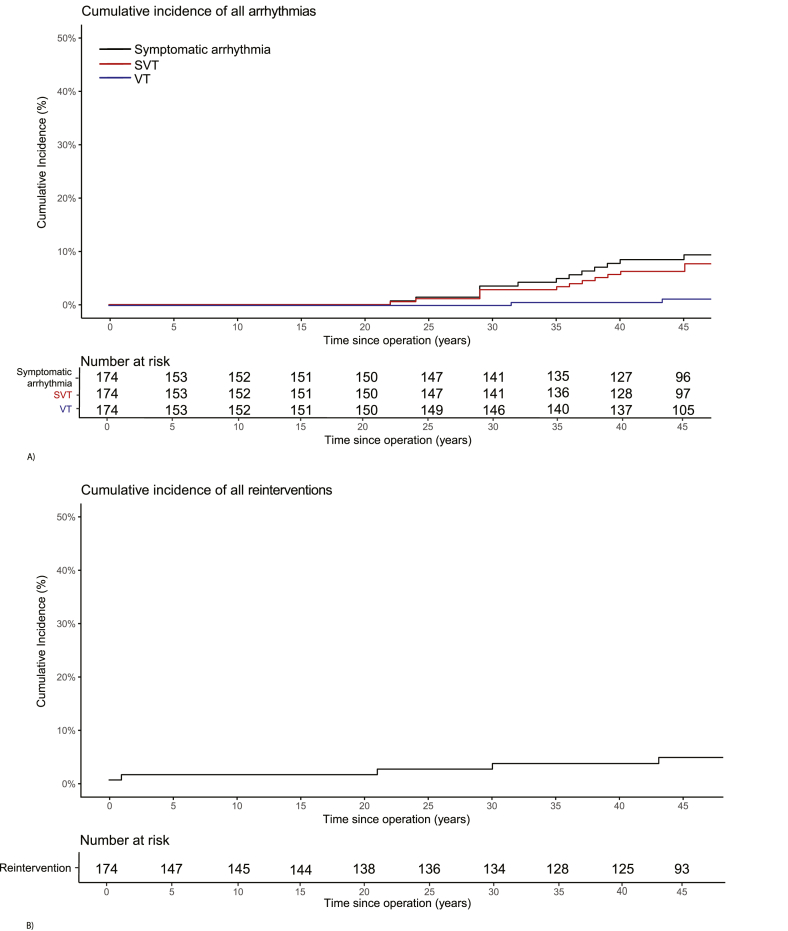
Cumulative incidence of symptomatic arrhythmias and cardiac reinterventions A) Cumulative incidence of symptomatic arrhythmia (at 49 years: 10 %, black), SVT (at 49 years: 9 %, red), and VT (at 49 years: 1 %, blue) B) Cumulative incidence of cardiac reintervention (at 49 years: 3 %, black) Panel A shows the cumulative incidence of symptomatic arrhythmias in the VSD cohort. After 49 years of follow-up, the overall incidence of symptomatic arrhythmias reached 10 %, with SVT accounting for 9 % and VT for 1 %. Panel B displays the cumulative incidence of VSD-related interventions. At 49 years, the cumulative incidence was 3 %.

Cumulative incidence of symptomatic arrhythmias was 10 % at 49 years (Fig. 3A). In the last decade, 6 patients developed new symptomatic arrhythmias (all 6 supraventricular and 1 also ventricular). Cumulative incidence of pacemaker implantation at 49 years was 8 %, with 3 pacemakers being implanted in the last decade. One patient developed bradycardia with slow junctional escape rhythm. Another patient developed complete AV block after redo-surgery (VSD patch replacement and tricuspid valve reconstruction after endocarditis). In the third patient, the indication was rhythm and conduction disturbances. Two patients received an ICD, both for secondary prevention after an out-of-hospital cardiac arrest. The first patient experiencing ventricular fibrillation had extensive coronary artery disease. The second patient had no identifiable substrate other than the VSD closure.

Cumulative incidence of VSD-related reintervention at 49 years of follow-up was 3 % (Fig. 3B). In the last decade, 1 patient suffered a stroke. He was known with a history of atrial flutter. No patient was diagnosed with heart failure or endocarditis.

### 3.4 In-hospital assessment

ECG and Holter findings are summarized in Table 2 and Supplementary Fig. 3. Sinus rhythm was present in 91 % of the patients. There was a significant increase in PR interval and QRS duration over time (both p < 0.001), with 14 % of patients having a PR interval >200 ms and 34 % having a QRS duration >120 ms at last follow-up.

**Table 2:** Diagnostic measurements.

	1990 (n = 107)	2001 (n = 95)	2012 (n = 91)	2022 (n = 76)	P for trend^a^
**Electrocardiography**	107	95	79	65	
Rhythm					0.58^b^
Sinus	104 (97 %)	86 (91 %)	72 (91 %)	59 (91 %)	
Atrial	1 (1 %)	2 (2 %)	2 (3 %)	1 (2 %)	
Nodal	1 (1 %)	3 (3 %)	1 (1 %)	0 (0 %)	
Pacemaker	1 (1 %)	4 (4 %)	4 (5 %)	4 (6 %)	
Atrial flutter	0 (0 %)	0 (0 %)	0 (0 %)	0 (0 %)	
Other	0 (0 %)	0 (0 %)	0 (0 %)	1 (1 %)	
Heart rate, bpm	73 ± 14	71 ± 15	65 ± 11	69 ± 14	<0.001
PR interval, ms	148 ± 29	151 ± 28	159 ± 30	167 ± 34	<0.001
PR > 200 ms	5 (5 %)	7 (8 %)	6 (8 %)	9 (14 %)	0.005
QRS duration, ms	101 ± 21	113 ± 27	117 ± 27	119 ± 28	<0.001
QRS duration >120 ms	26 (25 %)	29 (32 %)	25 (33 %)	22 (34 %)	0.10
					
**24-h Holter**	103	92	68	53	
Mean heart rate, bpm	89 ± 9	88 ± 10	88 ± 12	73 ± 8	<0.001
Supraventricular arrhythmias^c^	21 (20 %)	25 (27 %)	25 (37 %)	20 (38 %)	0.003
SND^d^	21 (20 %)	8 (9 %)	4 (6 %)	4 (8 %)	<0.001
Paroxysmal AF/AFL	0 (0 %)	0 (0 %)	1 (1 %)	0 (0 %)	NA
SVT 3–10 complexes	0 (0 %)	19 (21 %)	22 (32 %)	19 (36 %)	<0.001
SVT>10 complexes	0 (0 %)	0 (0 %)	4 (6 %)	5 (9 %)	0.007
VT 3–10 complexes	6 (6 %)	2 (2 %)	3 (4 %)	4 (5 %)	0.30
VT > 10 complexes	0 (0 %)	0 (0 %)	0 (0 %)	0 (0 %)	NA
					
**CPET**	103	93	69	51	
Maximal heart rate, %	89 ± 9	88 ± 10	88 ± 12	86 ± 13	<0.001
Maximum workload, %	94 ± 19	91 ± 24	87 ± 19	93 ± 26	0.02
Maximal exercise capacity <85 %	30 (29 %)	37 (40 %)	33 (48 %)	17 (33 %)	0.06
VO_2_ max, %			87 ± 20	87 ± 20	0.49
VO_2_ max <85 %			17 (25 %)	25 (49 %)	0.78
RER max				1.1 ± 0.1	
Arrhythmia	5 (5 %)	10 (11 %)	5 (7 %)	18 (35 %)	0.001
					
**Echocardiographic parameters**	107	95	76	60	
Normal systolic LV function	82 (81 %)	82 (86 %)	61 (79 %)	47 (78 %)	0.54
Normal systolic RV function	107 (100 %)	94 (99 %)	63 (83 %)	42 (70 %)	<0.001
LA end-systolic dimension, mm	32 ± 5	37 ± 6	38 ± 6	39 ± 6	<0.001
LA dilation^e^	1 (1 %)	9 (9 %)	21 (28 %)	35 (58 %)	<0.001
LV end-diastolic dimension, mm	49 ± 6	52 ± 5	50 ± 6	52 ± 7	0.006
LV dilation^f^	4 (4 %)	4 (4 %)	8 (11 %)	13 (22 %)	<0.001
Aorta end-diastolic dimension, mm	30 ± 5	33 ± 5	33 ± 6	36 ± 5	<0.001
Valve regurgitation (>trace)					
AR	12 (11 %)	16 (17 %)	16 (21 %)	15 (25 %)	0.52
MR	14 (13 %)	11 (12 %)	6 (8 %)	7 (12 %)	0.92
PR	23 (22 %)	27 (28 %)	8 (11 %)	7 (12 %)	0.11
TR	38 (38 %)	54 (57 %)	35 (47 %)	24 (40 %)	0.35
LV EF, %^g^^,^^h^			54 [49–61]	56 [53–60]	0.29
LV E/A ratio^h^			1.3 [1.1–1.7]	1.3 [1.0–1.6]	0.79
LV E/E′ ratio^h^			8.5 [6.3–10.0]	8.8 [7.3–11.7]	0.52
LV DET, ms^h^			185 [158–218]	195 [172–221]	0.34
Diastolic dysfunction^i^			8 (11 %)	24 (40 %)	<0.001
TAPSE, mm			19 [[18], [19], [20], [21], [22]]	19 [[17], [18], [19], [20], [21]]	0.36
RV FAC, %			40 [37–43]	43 [41–47]	0.40
Estimated RV systolic pressure, mmHg			28 [24–35]	28 [[24], [25], [26], [27], [28], [29], [30], [31]]	0.95

AF = atrial fibrillation; AFL = atrial flutter; AR = aortic regurgitation; DET = deceleration time; EF: ejection fraction; FAC = fractional area change; LA = left atrial; LV = left ventricular; MR = mitral regurgitation; PR = pulmonary regurgitation; RA, right atrial; RV = right ventricular; SND = sinus node disease; SVT = supraventricular tachycardia; TAPSE = tricuspid annular plane systolic excursion; TR = tricuspid regurgitation; Vmax = maximal velocity found with Doppler echocardiography; VO_2_max = *maximum rate of oxygen consumption attainable during physical exertion;* VT = ventricular tachycardia.

a Derived from (generalized) linear mixed models.

b Sinus rhythm vs other rhythms.

c Some patients had more than one type of supraventricular arrhythmia.

d Surface ECG Criteria for Sinus Node Dysfunction according to the Kugler criteria.

e Based on left atrial diameter and left atrial volume index.

f Based on left ventricular end-diastolic and end-systolic diameter.

g 2012 2D LVEF using the Simpson method vs 2022 3D LVEF.

h Additional diagnostic tests performed only in 2012, n = 38–72.

i Based on left ventricular E/A ratio, E/E′ ratio and deceleration time (echocardiography doppler).

During the 24-h Holter measurement, 20 (38 %) patients experienced short episodes of supraventricular arrhythmias, of which 19 (36 %) were supraventricular tachycardia (SVT), showing a significant increase over time (p < 0.001). Four patients had episodes of non-sustained ventricular tachycardia (VT) of 3–10 complexes. No atrial fibrillation or flutter was observed, neither were episodes of VT > 10 complexes, ventricular standstill or >3 s or total AV-block.

The CPET findings revealed that 33 % of patients had a reduced exercise capacity (<85 % of expected) and 49 % exhibited a reduced VO_2_max, ranging from 75 to 85 % of expected. Patients with nonisolated VSD had a lower VO_2_max than those with isolated VSD (72 % vs. 90 %, p = 0.03) but the exercise capacity was not significantly different (82 % vs. 95 %, p = 0.20).

Echocardiography showed an increase in left atrial (LA) end-systolic, LV end-diastolic and aortic dimensions over time (p < 0.001, 0.006 and < 0.001 respectively), and LA and LV dilation were present in 58 % and 22 % of the patients. Three patients showed signs of elevated pulmonary pressure (age at surgery: 3, 4 and 10 years) with a maximum tricuspid regurgitant jet velocity of 3.8 m/s or maximum pulmonary regurgitant jet velocity of 3 m/s.

CMR analysis showed LV end-diastolic volume dilation in 11 patients (22 %) and LV end-systolic volume dilation in 26 patients (53 %) (Table 3). The median LV ejection fraction (LVEF) was 55 % and 1 patient (1 %) had a LVEF <45 %. Five patients (10 %) had a small residual VSD, with 2 of the 5 also showing mild aortic regurgitation.

**Table 3:** Cardiac Magnetic Resonance imaging analysis.

2022	N ((%))	Median	25th-75th percentile
LV			
EDVi (mL/m^2^)	48	91	[80–102]
ESVi (mL/m^2^)	48	41	[34–47]
EF (%)	48	55	[52–59]
EF diminished	9 (19 %)		
EF<45 %	1 (1 %)		
RV			
EDVi (mL/m^2^)	48	95	[82–106]
ESVi (mL/m^2^)	48	44	[36–49]
EF (%)	48	53	[50–57]
EF diminished	2 (4 %)		
EF<45 %	3 (6 %)		

EDVi = end-diastolic volume index; EF = ejection fraction; ESVi = end-systolic volume index; LV = left ventricular; RV = right ventricular.

Laboratory measurements are reported in Supplementary Table 5. Median NT-proBNP level was 13.0 [7.3–20.5] pmol/L and elevated in 44 % of the patients. No correlation was found with other CMR-derived dimensions and volumes.

**Fig. 4:**
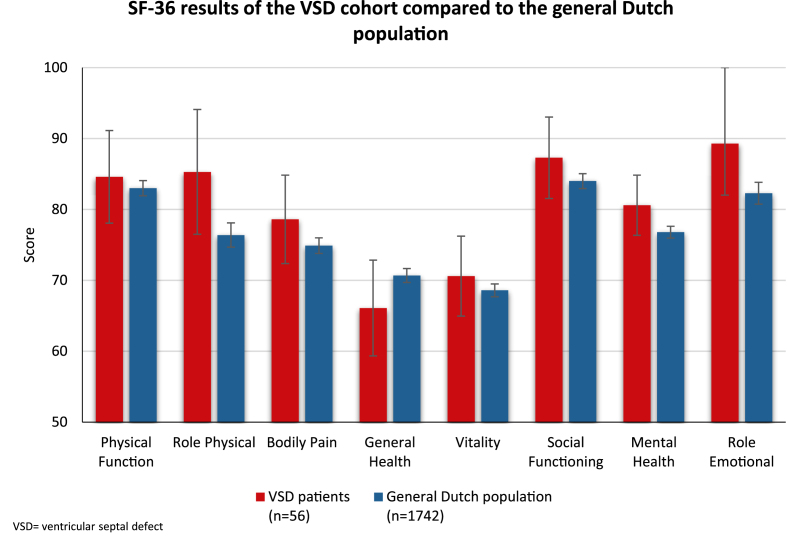
36-Item Short Form Survey results of the VSD cohort compared to the general Dutch population This figure presents the SF-36 health survey results of the VSD cohort in comparison to normative data from the general Dutch population. Scores across all domains were largely comparable, with no statistically significant differences observed.

Mean scores of the SF-36 survey for patients and age-matched GDP are shown in Fig. 4, Supplementary Figs. 4 and 5, and Supplementary Table 6. Results were comparable with the GDP. Over time, VSD patients scored significantly lower for the domains physical function and general health (p = 0.01 and p < 0.001 respectively). The outcomes were not significantly different between the patients with nonisolated and isolated VSD.

### 3.5 Predictors of clinical outcome

Results of the Cox regression analysis relating baseline parameters to clinical outcome are presented in Table 4. Early postoperative arrhythmias were found to predict all-cause mortality (Hazard Ratio (HR) = 8.62, 95 % confidence interval (CI) [4.05–18.39], p < 0.001). Joint models showed a statistically significant association between serially measured LVF over time and symptomatic arrhythmia (HR = 1.44, 95 % CI [1.06–2.36], p = 0.01).

**Table 4:** Predictor analyses of all-cause mortality and arrhythmias.

End Point^a^	Univariable Model			Multivariable Model		
	HR	95 % CI	P-value	HR	95 % CI	P-value
All-cause mortality (n = 39)						

Age at operation	0.98	[0.91–1.05]	0.54			
Operated after 1975	1.12	[0.59–2.14]	0.73			
Hypothermia during surgery	1.90	[0.996–3.63]	0.052			
Aortic clamp time (per 5 min)	1.14	[1.02–1.27]	**0.02**	1.12	[0.98–1.27]	0.09
LA saturation	0.93	[0.84–1.03]	0.14			
Early postoperative arrhythmias	7.80	[3.93–15.48]	**<0.001**	8.62	[4.05–18.39]	**<0.001**
Nonisolated VSD	1.71	[0.90–3.22]	0.10			

Arrhythmias (n = 14)						

Age at operation	0.96	[0.84–1.10]	0.58			
Operated after 1975	2.19	[0.65–7.31]	0.20			
Hypothermia during surgery	0.58	[0.16–2.12]	0.41			
Aortic clamp time (per 5 min)	1.20	[0.97–1.48]	0.10			
LA saturation	1.24	[0.86–1.78]	0.24			
Early postoperative arrhythmias^b^	NA	NA	NA			
Nonisolated VSD	1.24	[0.41–3.75]	0.71			

a Cox regression.

b The number of events is low and the available data is limited, Fisher's Exact Test: p = 0.37.

## 4 Discussion

In this study, survival and clinical outcomes of an unbiased cohort of patients with VSD who underwent surgical VSD closure at young age are described. These patients have been followed since their surgical closure every decade for almost 50 years. There is a notable lack of comprehensive literature on mortality, morbidity, predictor analyses, and QoL in patients after surgical VSD closure, both in the short- and long-term. The regular assessments, conducted every decade, allowed describing long-term outcome in an unbiased cohort, monitoring for changes in clinical outcomes and cardiac function over time, as well as identifying predictors of late outcome, especially mortality.

Our cohort included patients treated between 1968 and 1980, when surgical closure was the only available option for VSD. Historical data indicate low operative mortality and favorable long-term outcomes [3,13]. In contemporary cohorts, both surgical and transcatheter closure can be performed with lower risk and improved long-term outcomes [14,15]. These developments emphasize that outcomes in modern VSD patients may be better than those observed in historical cohorts.

### 4.1 Mortality

Cumulative survival at 49 years was 77 %, which is significantly lower than in the GDP. Within the first 30 days after surgery, 16 patients succumbed to either low cardiac output, cardiac tamponade, or conduction disorders. Later on, mortality was primarily due to SCD, malignancies or infections. In some cases, the cause was unknown, but SCD might be suspected, because these patients were relatively young, especially as 2 other patients suffered an out-of-hospital cardiac arrest, but were successfully resuscitated. Previous research reported survival rates ranging from 70 to 89 % at 40–60 years of follow-up [[15], [16], [17]].

Event-free survival at 49 years was 50 %, highlighting an unexpectedly high long-term morbidity associated with surgical VSD closure, especially symptomatic arrhythmias and pacemaker implantation. Event-free survival for patients with nonisolated vs. isolated VSD was 24 % and 62 %, respectively. Eighteen patients underwent intervention, of which 6 were dedicated to the VSD (2 in nonisolated and 4 in isolated VSD patients). The remaining events were primarily death (n = 36), SVT (n = 14) and pacemaker implantation (n = 10). These findings underscore that the potential challenges faced by VSD patients after surgical closure should not be underestimated, even though it is often viewed as a simple CHD.

### 4.2 Arrhythmias and sudden cardiac death

Cumulative incidence of symptomatic arrhythmias in our cohort was 10 % at 49 years, with 6 new cases in the last decade. SVT was the most prevalent arrhythmia, and the incidence of SVT increased with age [18,19]. VT was relatively rare, occurring in only 1 %. Importantly, 2 other patients suffered an out-of-hospital cardiac arrest and received an ICD: one with a Chronic Total Occlusion of the Left Anterior Descending artery, and the other likely due to surgical scarring in a complex VSD patient. Notably, the incidence of total AV block and the subsequent need for pacemaker implantation was significant. A total of 12 patients required pacemaker implantation, 9 of whom were not directly post-surgery. In the last decade, 3 patients required pacemaker implantation. This highlights the need for awareness of the increased risk of developing conduction disturbances in VSD patients later in life.

The location of the VSD has been found to play a crucial role in the risk of AV block, with inlet VSDs being associated with the highest risk [20]. Given the limited surgical options at the time, injury to the conduction system, and the associated risk of AV block, could not always be prevented. In addition ventriculotomy is known to predispose to other rhythm disturbances, such as right bundle branch block and ventricular arrhythmias [21]. These arrhythmias tend to increase over time, particularly with older age at surgery. In our cohort, both patients with event VT and those with non-sustained VT detected on Holter monitoring had a Dacron patch in place. Additionally, pulmonary artery (PA) banding was sometimes performed before surgical VSD closure to manage pulmonary hypercirculation. However, PA banding is known to lead to RV hypertrophy, increased RV pressure, and fibrosis, factors that significantly elevate the long-term risk of arrhythmias [22]. Since the number of patients in our cohort who underwent PA banding before surgery was low, we could not evaluate this. In current practice, VSD closure is typically performed via a transatrial approach, often at younger age, with PA banding rarely done. The shift away from PA banding and right ventriculotomy, alongside advancements in surgical techniques, has likely contributed to better short- and long-term outcomes for patients undergoing VSD closure nowadays [15].

Unexpectedly, 3 patients in our cohort died from SCD and 9 from unknown causes. In addition, 2 patients survived an out-of-hospital cardiac arrest. Previous research confirmed this, indicating that a higher SCD risk in surgically closed VSD patients, compared to age-matched controls, is to be expected [23]. The occurrence of (early postoperative) arrhythmia or a conduction disorder could serve as an important marker for identifying patients at higher risk of future complications, including SCD. This was even observed in 5 patients with isolated VSD. Although VSD is often considered a simple CHD, our findings emphasize the significant long-term arrhythmic and electrical complications that can arise in this population. Therefore, in symptomatic patients, routine Holter monitoring is indicated, and in selected cases, the use of implantable loop recorders for extended rhythm monitoring should be considered [24].

### 4.3 Reintervention

Cumulative incidence of reintervention for VSD at 49 years was only 3 %. This confirms the effectiveness of surgical VSD closure techniques, with minimal need for reoperation.

### 4.4 Heart failure and ventricular function

Cumulative incidence of heart failure was 3 % with no new cases in the last decade. Both LVEF and RVEF remained stable over time, with just 1 % and 6 % of patients showing an EF below 45 % on CMR. Therefore, we can conclude that heart failure and significant reduction in EF are rare in the long term after surgical VSD closure. Aortic regurgitation, another feared finding in this patient population, was seen often, but it is reassuring that all patients in our cohort exhibited only mild regurgitation with no substantial increase over time. The observed increase in LA end-systolic, LV end-diastolic, and aortic dimensions over time, as well as the LA and LV dilation, are partly related to the natural aging process [19]. Structural and functional changes in the heart, including increased vascular stiffness, left ventricular wall thickening, and fibrosis, typically occur with aging. Despite these age-related structural changes, the systolic ventricular function generally remained stable.

Interestingly, NT-proBNP levels were elevated in 45 % of patients, despite the majority being asymptomatic (91 % NYHA class I) with stable systolic biventricular function. However, in most cases the NT-proBNP level was only mildly elevated. No association with relevant medical history or clinical events could be identified. Sixteen of these patients exhibited systemic arterial hypertension and/or signs of diastolic dysfunction. These observations suggest that the mildly elevated NT-proBNP levels were likely due to these factors or aging, rather than being directly related to the previously closed VSD [25,26].

### 4.5 Other complications

Cumulative incidence of endocarditis was 3 %. Fortunately, no new cases were identified over the past decade. A total of 5 patients were diagnosed, 2 of whom had an infected patch, and 1 of whom also had a residual VSD. Cumulative incidence of stroke was 1 %, with only one event occurring in the past decade. This is consistent with existing research, which suggests that the incidence rate of ischemic stroke is low in VSD patients [27].

### 4.6 Predictors of clinical outcome

Our findings identified early postoperative arrhythmias as an independent predictor for mortality, which is consistent with clinical expectations and aligns with our previous findings [3]. Arrhythmias are known to complicate recovery and increase the risk of adverse outcomes, particularly when not properly managed. However, it is important to consider whether these arrhythmias are truly an independent predictor of poor outcomes, or if they serve as an indicator of underlying cardiac dysfunction or deterioration. Our results suggest that aorta clamp time and hypothermia appear to play a role, with longer aorta clamp times potentially leading to more ischemia and increased mortality, while hypothermia may increase the risk of arrhythmias and hemodynamic instability. However, statistical significance was not achieved. Additionally, we demonstrated a significant association between serially measured LVF and symptomatic arrhythmias. Although we did not identify conduction disorders as a risk factor in our study, another study has shown that transient and complete heart blocks are associated with late mortality [28]. This reinforces the importance of being aware of the potential link between AV block, pacemaker dependency, and mortality over the course of a VSD patient's lifetime.

### 4.7 Exercise capacity and quality of life

At the latest follow-up, VSD patients showed good self-reported QoL scores, comparable to the GDP. However, physical function and general health declined over time. This was especially the case in nonisolated VSD patients and therefore probably not caused by the VSD itself, but by their additional lesions. In addition, we might suggest that those patients with more complex CHD received more restrictive advices. Patients with isolated VSD showed good QoL scores and relatively preserved exercise capacity, and although both QoL and exercise capacity tended to be better in this group, the differences compared to nonisolated VSD patients were not statistically significant. Other studiy has also reported that patients who underwent VSD closure report lower physical functioning and general health scores [29]. Surgical intervention does not completely overcome the physical challenges that these patients experience. Interestingly, a study investigating the distribution of exercise capacity across the adults with CHD (ACHD) spectrum found that VSD patients typically exhibit only mild impairments in VO_2_max like in our study [30]. It is possible that many of these patients were advised in childhood to limit sports and physical exertion due to their condition, especially in children who were protectively nurtured by their parents [31].

### 4.8 Indications for follow-up

The need for long-term follow-up in patients with VSD after surgical correction remains a topic of ongoing debate. While many patients may not require frequent visits, certain factors can significantly influence the decision to continue monitoring these patients. Key risk factors include arrhythmias and conduction disorders. Significant valve disease and LV dysfunction can exacerbate cardiovascular symptoms and further compromise the heart function. Additionally, patients with residual or additional lesions may require closer monitoring to ensure timely intervention and prevent complications.

The AHA-ACC guidelines recommend regular follow-up with an ACHD cardiologist, with visit frequency ranging from every 3–36 months [32]. The ESC guidelines highlight the risk of complete AV block, especially after bifascicular or transient trifascicular block, and recommend annual visits for high-risk patients with residual defects, valvular or hemodynamic issues [33]. Low-risk patients may be followed every 3–5 years. A survey comparing these guidelines recommends VSD patients receive cardiology visits every 5 years with ECG and echocardiography [4]. Future research should define the best follow-up strategy, but based on the current data some routine cardiology visits indeed seem indicated.

### 4.9 Study limitations

Given the limited sample size in this study, caution is warranted in interpreting the findings. However, this cohort represents, to the best of our knowledge, the longest available follow-up period and consists of a longitudinally studied, unbiased group of patients who underwent surgical VSD closure. Survival data were accessible for 95 % of the original cohort, with 85 % of eligible survivors included in the present study. Importantly, no significant differences were observed between participating and nonparticipating patients, which helps reduce potential selection bias. Despite this, the inevitable smaller sample size compared to previous evaluations limits the statistical power. It should be noted that surgery and its timing, myocardial protection, postoperative care, and follow-up strategies have evolved since this cohort was treated, including diagnostic methods, shifting definitions of heart failure, changes in procedural interventions such as pacemaker implantation and ablation, and advances in arrhythmia management and pharmacological therapy, which may limit the applicability of our findings to current practice. Additionally, as a single-center study, outcomes may have been influenced by institutional protocols, surgical approaches, and individual surgeon experience, which could limit generalizability. Lastly, upon reflection, some patients have been included who also had another CHD such as pulmonary stenosis/RV outflow tract obstruction. These patients were also included in the previous evaluation moments. Therefore, we decided to analyze the original cohort and made a separate analysis of patients with and without concomitant lesions.

## 5 Conclusion

Survival and event-free survival after surgical VSD correction in this cohort were 77 % and 50 % at 49 years. For patients with isolated VSD, cumulative survival was 80 % and event-free survival 62 %. Despite often being considered a simple CHD, the cumulative incidence of symptomatic arrhythmias in this cohort was 10 % and of conduction disorders requiring pacemaker implantation 8 % respectively. Early postoperative arrhythmias predicted mortality. QoL was good and remained stable. Importantly, patients without residual lesions and/or sequalae do not need continued frequent follow-up and can be reassured. With the advances in surgical techniques, outcomes are expected to be even better for patients undergoing VSD closure nowadays.

## CRediT authorship contribution statement

**Sahra Ünlütürk:** Writing – original draft, Visualization, Validation, Project administration, Methodology, Investigation, Formal analysis, Data curation, Conceptualization. **Robert M. Kauling:** Writing – original draft, Supervision, Methodology, Investigation, Data curation, Conceptualization. **Judith A.A.E. Cuypers:** Writing – review & editing, Methodology, Formal analysis, Conceptualization. **Annemien E. van den Bosch:** Writing – review & editing. **Alexander Hirsch:** Writing – review & editing, Formal analysis. **Chiara Pelosi:** Writing – review & editing, Formal analysis, Data curation. **Daniel J. Bowen:** Writing – review & editing, Formal analysis. **Raluca G. Chelu:** Writing – review & editing, Data curation. **Ad J.J.C. Bogers:** Writing – review & editing, Conceptualization. **Willem A. Helbing:** Writing – review & editing. **Isabella Kardys:** Writing – review & editing, Supervision, Methodology, Formal analysis. **Jolien W. Roos-Hesselink:** Writing – review & editing, Supervision, Project administration, Methodology, Funding acquisition, Formal analysis, Conceptualization.

## Funding

This work was supported by Stichting ‘De Hoop Leven’ and ‘Thoraxfoundation’.

## Declaration of competing interest

The authors declare that they have no known competing financial interests or personal relationships that could have appeared to influence the work reported in this paper other t
