# Supplementary material for: Long-term outcome after surgical ventricular septal defect closure: Longitudinal follow-up into the fifth decade
Source: Int J Cardiol Congenit Heart Dis. 2025 Oct 10;22:100624. doi: 10.1016/j.ijcchd.2025.100624 (PMC12594936; doi:10.1016/j.ijcchd.2025.100624)
Supplement: Multimedia component 1 [file mmc1.docx]

**Supplementary Table 1.** Vendor and reference values of the used laboratory measurements.

| **Laboratory measurement** | **Vendor** | **Reference value** |
| --- | --- | --- |
| Creatinine | Cobas 8000, Roche diagnostics, Basel Switzerland. | male 65-115 umol/L, female 55-90 umol/L |
| NT-proBNP | Cobas 8000, Roche diagnostics, Basel Switzerland. | < 15 pmol/L |
| HS-Troponin T | Cobas 8000, Roche diagnostics, Basel Switzerland. | <14 ng/L |
| HS-CRP | Cobas 8000, Roche diagnostics, Basel Switzerland. | <10 mg/L |
| Total cholesterol | Cobas 8000, Roche diagnostics, Basel Switzerland. | 2.9-6.5 mmol/L |
| LDL cholesterol | Cobas 8000, Roche diagnostics, Basel Switzerland. | 2.59-4.12 mmol/L |
| HDL cholesterol | Cobas 8000, Roche diagnostics, Basel Switzerland. | >1.55 mmol/L |
| Triglycerides | Cobas 8000, Roche diagnostics, Basel Switzerland. | <2.0 mmol/L |
| HbA1c | Menarine diagnostics, Florence Italy | 26-42 mmol/mol Hb |
| TSH | Lumipulse, Fujirebio diagnostics, Malvern USA | 0.56-4.27 mU/L |
| FT4 | Lumipulse, Fujirebio diagnostics, Malvern USA | 13.5-24.3 pmol/L |
| Hb | XN 9000, Sysmex diagnostics, Hamburg Germany | male 8.6-10.5 mmol/L, female 7.5-9.5 mmol/L |

CRP= C-reactive protein; FT4= free thyroxine; Hb= haemoglobin; HbA1C= Haemoglobin A1C; HDL= high-density lipoprotein; LDL= low-density lipoprotein; NT-proBNP= N-terminal prohormone of brain natriuretic peptide; TSH= thyroid stimulating hormone

**Supplementary Table 2A.** Participants vs. non-participants

|  | **Participation 2022** | **No participation 2022** | **P-value** |
| --- | --- | --- | --- |
|  | **N=76** | **N=98** |  |
| Male | 45 (59%) | 51 (52%) | 0.35 |
| Age at operation, years | 3.1 [0.6-6.5] | 2.0 [0.4-7.1] | 0.62 |
| Age at operation <1 year | 24 (32%) | 36 (37%) | 0.48 |
| Pre-operative RV systolic pressure, mmHg | 68 [42-80] | 70 [52-85] | 0.25 |
| Pre-operative Qp/Qs ratio | 2.0 [1.7-2.8] | 2.1 [1.6-2.8] | 0.95 |
| Type of VSD |  |  | 0.39 |
| Perimembranous | 60 (79%) | 74 (76%) |  |
| Muscular | 5 (7%) | 3 (3%) |  |
| Nonisolated VSD^†^ | 19 (25%) | 38 (39%) | 0.06 |
| Patent foramen ovale/  atrial septal defect | 11 (58%) | 14 (37%) |  |
| Patent ductus arteriosus | 4 (21%) | 10 (26%) |  |
| Coarctation aortae | 4 (21%) | 5 (13%) |  |
| Pulmonary stenosis | 7 (37%) | 8 (21%) |  |
| Mitral stenosis | 1 (5%) | 1 (3%) |  |
| Other | 1 (5%) | 7 (18%) |  |
| Previous PA banding | 4 (5%) | 12 (12%) | 0.11 |
| Hypothermia |  |  | 0.29 |
| Temperature <20°C | 28 (37%) | 44 (45%) |  |
| Temperature 20-35°C | 47 (62%) | 53 (54%) |  |
| Temperature unknown | 1 (1%) | 1 (1%) |  |
| RV incision | 39 (51%) | 39 (40%) | 0.30 |
| VSD closure with patch | 64 (84%) | 84 (86%) | 0.10 |
| Post-operative arrhythmia <30 days | 2 (3%) | 14 (17%) | 0.03 |
| Heart block <30 days | 2 (3%) | 7 (7%) | NA |
| Follow-up, years | 44 [43-46] |  |  |
| Age at study, years | 49 [44-54] |  |  |

|  | **Participation 2022** | **No participation 2022^*^** | **P- value** |
| --- | --- | --- | --- |
|  | **N=76** | **N=50** |  |
| Male | 45 (59%) | 27 (54%) | 0.56 |
| Age at operation, years | 3.1 [0.6-6.5] | 2.3 [0.6-7.3] | 0.91 |
| Age at operation <1 year | 24 (32%) | 15 (30%) | 0.85 |
| Pre-operative RV systolic pressure, mmHg | 68 [42-80] | 66 [53-85] | 0.62 |
| Pre-operative Qp/Qs ratio | 2.0 [1.7-2.8] | 2.1 [1.7-2.7] | 0.90 |
| Type of VSD |  |  | 0.64 |
| Perimembranous | 60 (79%) | 39 (78%) |  |
| Muscular | 5 (7%) | 3 (6%) |  |
| Nonisolated VSD^†^ | 19 (25%) | 17 (34%) | 0.27 |
| Patent foramen ovale/  atrial septal defect | 11 (58%) | 5 (29%) |  |
| Patent ductus arteriosus | 4 (21%) | 6 (35%) |  |
| Coarctation aortae | 4 (21%) | 1 (6%) |  |
| Pulmonary stenosis | 7 (37%) | 2 (12%) |  |
| Mitral stenosis | 1 (5%) | 0 (0%) |  |
| Other | 1 (5%) | 3 (18%) |  |
| Previous PA banding | 6 (8%) | 4 (8%) | 0.71 |
| Hypothermia |  |  | 0.76 |
| Temperature <20°C | 28 (37%) | 20 (40%) |  |
| Temperature 20-35°C | 47 (62%) | 30 (60%) |  |
| Temperature unknown | 1 (1%) | 0 (0%) |  |
| RV incision | 39 (51%) | 23 (46%) | 0.84 |
| VSD closure with patch | 64 (84%) | 42 (84%) | 0.28 |
| Post-operative arrhythmia <30 days | 2 (3%) | 1 (2%) | NA |
| Heart block <30 days | 2 (3%) | 2 (4%) | NA |
| Follow-up, years | 44 [43-46] |  |  |
| Age at study, years | 49 [44-54] |  |  |

PA= pulmonary artery; RV= right ventricle/ventricular; VSD= ventricular septal defect

*Exclusion of the deceased patients

^†^18 patients had more than one concomitant lesion besides ventricular septal defect

**Supplementary Table 2B.** Additional baseline characteristics VSD cohort last evaluation moment

|  | **2022** |
| --- | --- |
|  | **N=76** |
| Hypertension | 11 (14%) |
| Diabetes Mellitus II | 4 (5%) |
| Hypercholesterolemia | 3 (4%) |
| Smoking | 12 (16%) |
| Medication |  |
| None | 37 (49%) |
| ACE inhibitors/angiotensin receptor neprilysin inhibitors | 11 (14%) |
| Sacubitril/valsartan | 1 (1%) |
| Beta blockers | 8 (11%) |
| Calcium antagonists | 4 (5%) |
| Loop diuretics | 6 (8%) |
| Mineralcorticosteroids receptor antagonists | 0 (0%) |
| SGLT_2_ inhibitors | 1 (1%) |
| Oral anticoagulants | 11 (14%) |
| Aspirin | 3 (4%) |
| Antiarrhythmics | 3 (4%) |
| Digoxin | 0 (0%) |
| Oral nitrates | 1 (1%) |
| Cholesterol-lowering drugs | 5 (7%) |

**Supplementary Table 3.** Details regarding causes of death.

| **Patient (sex/ age at death)*** | **Follow-up until death (years)** | **Type of VSD** | **(Non)isolated VSD** | **Cause of death** |
| --- | --- | --- | --- | --- |
| Post-operative |  |  |  |  |
| Patient 1 (♀, 0 years old) | 0 | Other | Nonisolated | Low cardiac output |
| Patient 2 (♀, 0 years old) | 0 | Perimembraneous | Nonisolated | Cardiac arrest due to RV bleeding and cerebral lesions |
| Patient 3 (♀, 0 years old) | 0 | Perimembraneous | Nonisolated | Total AV block and drop intra-arterial pressure |
| Patient 4 (♂, 0 years old) | 0 | Other | Isolated | Cardiac arrest |
| Patient 5 (♂, 0 years old) | 0 | Perimembraneous | Nonisolated | Toxic shock from pseudomonas sepsis |
| Patient 6 (♂, 0 years old) | 0 | Muscular | Isolated | Bronchopneumonia and lung congestion |
| Patient 7 (♂, 0 years old) | 0 | Perimembraneous | Nonisolated | Cardiac arrest |
| Patient 8 (♂, 0 years old) | 0 | Perimembraneous | Isolated | Total AV block and asystole |
| Patient 9 (♀, 0 years old) | 0 | Perimembraneous | Nonisolated | Nodal and irregular rhythm, subsequently cardiac arrest and death, autopsy: severe fibroelastosis of LA and LV |
| Patient 10 (♀, 0 years old) |  | Perimembraneous | Nonisolated | Irreversible shock due to low cardiac output |
| Patient 11 (♀, 0 years old) | 0 | Perimembraneous | Isolated | Pulmonary emboli and organ infarcts |
| Patient 12 (♀, 0 years old) | 0 | Muscular | Isolated | Tamponade and brain death |
| Patient 13 (♀, 0 years old) | 0 | Perimembraneous | Nonisolated | Massive bleeding, heart lacerations, and total AV block |
| Patient 14 (♂, 0 years old) | 0 | Other | Isolated | Cerebral edema and brainstem entrapment |
| Patient 15 (♀, 0 years old) | 0 | Perimembraneous | Isolated | Poor cardiac recovery post-surgery |
| Patient 16 (♀, 0 years old) | 0 | Muscular | Isolated | Cardiac tamponade, malignant tachycardia, and low cardiac output |
| Other |  |  |  |  |
| Patient 17 (♀, 0 years old) | 0 | Muscular | Isolated | Autopsy: left atrial fibroelastosis, ischemic necrosis, and fibrosis of the liver and kidneys due to severe hypoxemia |
| Patient 18 (♂, 13 years old) | 0 | Perimembraneous | Isolated | Staphylococcal sepsis and endocarditis of the aortic and tricuspid valve |
| Patient 19 (♀, 3 years old) | 2 | Perimembraneous | Isolated | Unknown |
| Patient 20 (♀, 24 years old) | 13 | Perimembraneous | Nonisolated | Autopsy: fibrous pericarditis, subarachnoid haemorrhage on the left and around the brainstem, and cerebral edema |
| Patient 21 (♂, 17 years old) | 15 | Perimembraneous | Nonisolated | Died due to an accident with a deep fryer |
| Patient 22 (♀, 20 years old) | 16 | Perimembraneous | Isolated | Cardiac arrest, known with severe pulmonary hypertension |
| Patient 23 (♀, 23 years old) | 20 | Other | Nonisolated | Unknown |
| Patient 24 (♂, 28 years old) | 27 | Perimembraneous | Isolated | Deceased due to electromechanical dissociation in the context of severe DCM and severe secondary mitral insufficiency associated with PH |
| Patient 25 (♀, 41 years old) | 29 | Perimembraneous | Nonisolated | Deceased due to breast cancer with multiple brain metastases |
| Patient 26 (♂, 39 years old) | 31 | Perimembraneous | Isolated | Unknown, at the time admitted for ascites, jaundice, and high blood pressure |
| Patient 27 (♂, 40 years old) | 33 | Perimembraneous | Nonisolated | Metastasized small cell lung carcinoma |
| Patient 28 (♂, 34 years old) | 34 | Other | Isolated | Sudden cardiac death |
| Patient 29 (♀, 35 years old) | 34 | Perimembraneous | Nonisolated | Unknown |
| Patient 30 (♂, 49 years old) | 39 | Other | Isolated | Sudden cardiac death |
| Patient 31 (♂, 41 years old) | 40 | Other | Isolated | Thyroid carcinoma with metastases |
| Patient 32 (♂, 42 years old) | 42 | Perimembraneous | Isolated | Unknown |
| Patient 33 (♂, 45 years old) | 42 | Perimembraneous | Isolated | Malignant infarction of the left middle and anterior cerebral artery |
| Patient 34 (♂, 52 years old) | 42 | Perimembraneous | Nonisolated | Unknown |
| Patient 35 (♀, 51 years old) | 43 | Perimembraneous | Nonisolated | Sudden cardiac death |
| Patient 36 (♀, 52 years old) | 43 | Other | Isolated | Unknown |
| Patient 37 (♀, 53 years old) | 43 | Perimembraneous | Isolated | Unknown |
| Patient 38 (♂, 65 years old) | 51 | Other | Nonisolated | COVID pneumonia |
| Patient 39 (♂, age unknown) | Unknown | Perimembraneous | Isolated | Unknown |

AV= atrioventricular; DCM= dilated cardiomyopathy; LA= left atrium; LV= left ventricle; PH= pulmonary hypertension
*Results last evaluation moment

**Supplementary Table 4.** Total of first events

| First events* | Before 2012 | 2012-2022 | Total |
| --- | --- | --- | --- |
|  | N=174 | N=71 |  |
| Death | 31 | 8 | 39 |
| Intervention | 16 | 2 | 18 |
| Reintervention | 5 | 1 | 6 |
| Additional interventions | 11 | 1 | 12 |
| Symptomatic arrhythmias | 8 | 6 | 14 |
| SVT | 7 | 6 | 13 |
| VT | 1 | 1 | 2 |
| Pacemaker | 9 | 3 | 12 |
| ICD | 2 | 2 | 4 |
| Heart failure | 4 | 0 | 4 |
| CVA | 0 | 1 | 1 |
| Endocarditis | 5 | 0 | 5 |

CVA= cerebrovascular accident; ICD= implantable cardioverter defibrillator; SVT= supraventricular tachycardia; VT= ventricular tachycardia

*Some patients had more than 1 event

^†^Reintervention: residual VSD closure, aortic valve replacement due to aortic regurgitation, bicuspid aortic valve replacement

^#^Additional intervention: re-operation for patent ductus arteriosus, ballon dilatation or resection of (re)coarctation, correction or ballon dilatation of infundibular pulmonary stenosis, resection of subvalvular aortic stenosis, repair of ascending aorta aneurysm and Bentall procedure

**Supplementary Table 5.** Laboratory results

|  | **N** | **2022** |
| --- | --- | --- |
| Creatinine (umol/L) | 55 | 80.0 [69.0-93.0] |
| Creatinine abnormal^†^ |  | 6 (10%) |
| NT-proBNP (pmol/L) | **52** | **13.0 [7.3-20.5]** |
| NT-proBNP ≥15 pmol/L |  | **23 (44%)** |
| HS-Troponin T (ng/L) | 51 | 5.0 [4.0-8.0] |
| HS-Troponin T ≥14 ng/L |  | **4 (8%)** |
| Lipid spectrum |  |  |
| Total cholesterol (mmol/L) | 51 | 5.1 [4.6-5.7] |
| Total cholesterol abnormal^#^ |  | 3 (6%) |
| LDL cholesterol (mmol/L) | 51 | 3.4 [2.7-3.9] |
| LDL cholesterol abnormal^$^ |  | **19 (37%)** |
| HDL cholesterol (mmol/L) | 51 | 1.4 [1.2-1.8] |
| HDL cholesterol ≤1.55 mmol/L |  | **29 (57%)** |
| Triglycerides (mmol/L) | 51 | 1.1 [0.9-1.6] |
| Triglycerides ≥2 mmol/L |  | 8 (16%) |
| CRP (mg/L) | 55 | 1.2 [0.6-2.2] |
| CRP ≥10 mg/L |  | 3 (6%) |
| Hb (mmol/L) | 54 | 9.0 [8.6-9.5] |
| Hb abnormal^÷^ |  | 2 (4%) |
| TSH (mU/L) | 51 | 1.8 [1.2-2.6] |
| TSH abnormal^∆^ |  | 2 (4%) |
| FT4 (pmol/L) | 51 | 17.4 [16.0-19.3] |
| FT4 abnormal^€^ |  | 2 (4%) |
| HbA1c (mmol/mol) | 51 | 36.0 [34.0-38.0] |
| HbA1c abnormal^%^ |  | 3 (6%) |

CRP= C-reactive protein; FT4= free thyroxine; HbA1C= glycated haemoglobin ; Hb= haemoglobin; HDL= high-density lipoprotein; HS-Troponin-T= high-sensitivity cardiac troponin T; LDL= low-density lipoprotein; NT-proBNP= N-terminal pro b-type natriuretic peptide; TSH= thyroid stimulating hormone

**Supplementary Table 6.** 36-Item Short Form Survey results of the VSD cohort

|  |  | **VSD QoL3**  **(n=72)** | | | | | | **VSD QoL4 (n=56)** | | | | | | **Norm (n=1742)** | | | | | | **P-value QoL4 vs QoL3** | | **P-value QoL4 vs GDP** | | | |
| --- | --- | --- | --- | --- | --- | --- | --- | --- | --- | --- | --- | --- | --- | --- | --- | --- | --- | --- | --- | --- | --- | --- | --- | --- | --- |
| Physical complex | | | | | | | | | | | | | | | | | | | | | | |  |  |  |
| Physical Function | RP | 90.3 | | ± | 17.2 | | | 84.6 | | ± | | 24.9 | | 83.0 | | ± | | 22.8 | | 0.08 | | 0.64 | | | |
| Role Physical | BP | 89.6 | | ± | 26.5 | | | 85.3 | | ± | | 33.6 | | 76.4 | | ± | | 36.3 | | 0.28 | | 0.05 | | | |
| Bodily Pain | SF | 86.2 | | ± | 20.2 | | | 78.6 | | ± | | 23.8 | | 74.9 | | ± | | 23.4 | | 0.07 | | 0.25 | | | |
| General Health | PF | 73.9 | | ± | 22.8 | | | 66.1 | | ± | | 25.8 | | 70.7 | | ± | | 20.7 | | **0.01** | | 0.19 | | | |
| Mental complex | | | | | | | | | | | | | | | | | | | | | | |  |  |  |
| Vitality | GH | 74.6 | | ± | 18.5 | | | 70.6 | | ± | | 21.5 | | 68.6 | | ± | | 19.3 | | 0.17 | | 0.48 | | | |
| Social Functioning | MH | 91.8 | | ± | 18.5 | | | 87.3 | | ± | | 21.9 | | 84.0 | | ± | | 22.4 | | 0.24 | | 0.27 | | | |
| Mental Health | RE | 87.2 | | ± | 32.9 | | | 80.6 | | ± | | 16.2 | | 76.8 | | ± | | 17.4 | | 0.33 | | 0.09 | | | |
| Role Emotional | VT | 93.1 | | ± | 18.5 | | | 89.3 | | ± | | 27.8 | | 82.3 | | ± | | 32.9 | | 0.27 | | 0.07 | | | |
|  |  | **VSD QoL4**  **Male (n=34)** | | | | | **VSD QoL4**  **Female (n=22)** | | | | | | **Norm (n=1742)** | | | | | | **P-value Male vs Norm** | | **P-value Female vs Norm** | | | **P-value Male vs Female** |  |
| Physical complex | | | | | | | | | | | | | | | | | | | | | | | | |  |
| Physical Function | PF | 82.2 | ± | | | 28.6 | 88.2 | | ± | | 18.0 | | 83.0 | | ± | | 22.8 | | 0.87 | | 0.19 | | | 0.34 |  |
| Role Physical | RP | 81.6 | ± | | | 38.6 | 90.9 | | ± | | 23.8 | | 76.4 | | ± | | 36.3 | | 0.44 | | **0.009** | | | 0.27 |  |
| Bodily Pain | BP | 78.2 | ± | | | 26.7 | 79.2 | | ± | | 18.9 | | 74.9 | | ± | | 23.4 | | 0.48 | | 0.30 | | | 0.87 |  |
| General Health | GH | 64.0 | ± | | | 27.4 | 69.3 | | ± | | 23.4 | | 70.7 | | ± | | 20.7 | | 0.16 | | 0.79 | | | 0.44 |  |
| Mental complex | | | | | | | | | | | | | | | | | | | | | | | | |  |
| Vitality | VT | 70.0 | ± | | | 23.6 | 71.6 | | ± | | 18.2 | | 68.6 | | ± | | 19.3 | | 0.73 | | 0.45 | | | 0.78 |  |
| Social Functioning | SF | 83.8 | ± | | | 25.7 | 92.6 | | ± | | 13.2 | | 84.0 | | ± | | 22.4 | | 0.97 | | **0.006** | | | 0.10 |  |
| Mental Health | MH | 79.3 | ± | | | 16.7 | 82.5 | | ± | | 15.6 | | 76.8 | | ± | | 17.4 | | 0.39 | | 0.10 | | | 0.46 |  |
| Role Emotional | RE | 87.3 | ± | | | 30.7 | 92.4 | | ± | | 22.8 | | 82.3 | | ± | | 32.9 | | 0.35 | | 0.05 | | | 0.47 |  |

Norm= general Dutch population; QoL3= Quality of Life 3; QoL4= Quality of Life 4; VSD= Ventricular Septal Defect

**Supplementary Figure 1.** Cumulative survival and event-free survival of nonisolated vs isolated VSD patients

| 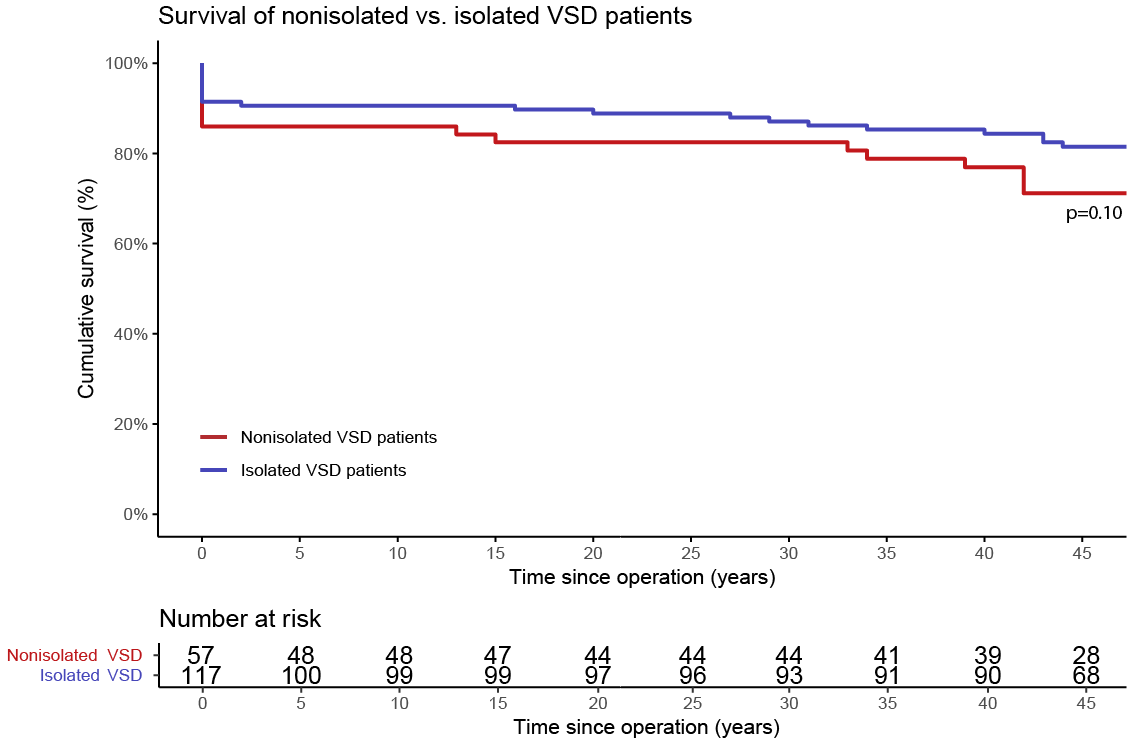A) Survival plot of the patients with nonisolated (at 49 years: 71%, red) and isolated VSD (at 49 years: 81%, blue) |
| --- |
| 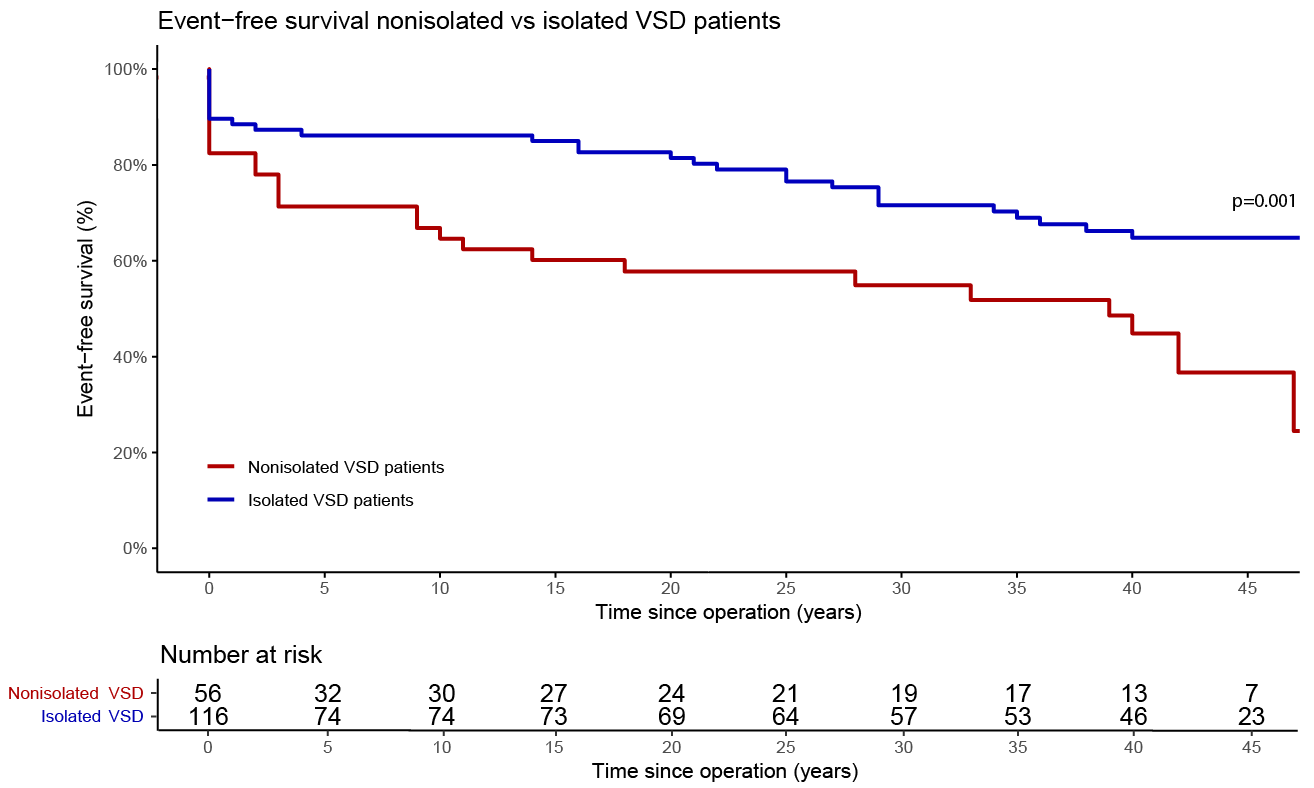B) Event-free survival of the patients with nonisolated (at 49 years: 24%, red) and isolated VSD (at 49 years: 62%, blue) |

**Supplementary Figure 2.** Cumulative survival and event-free survival of male and female VSD patients

| 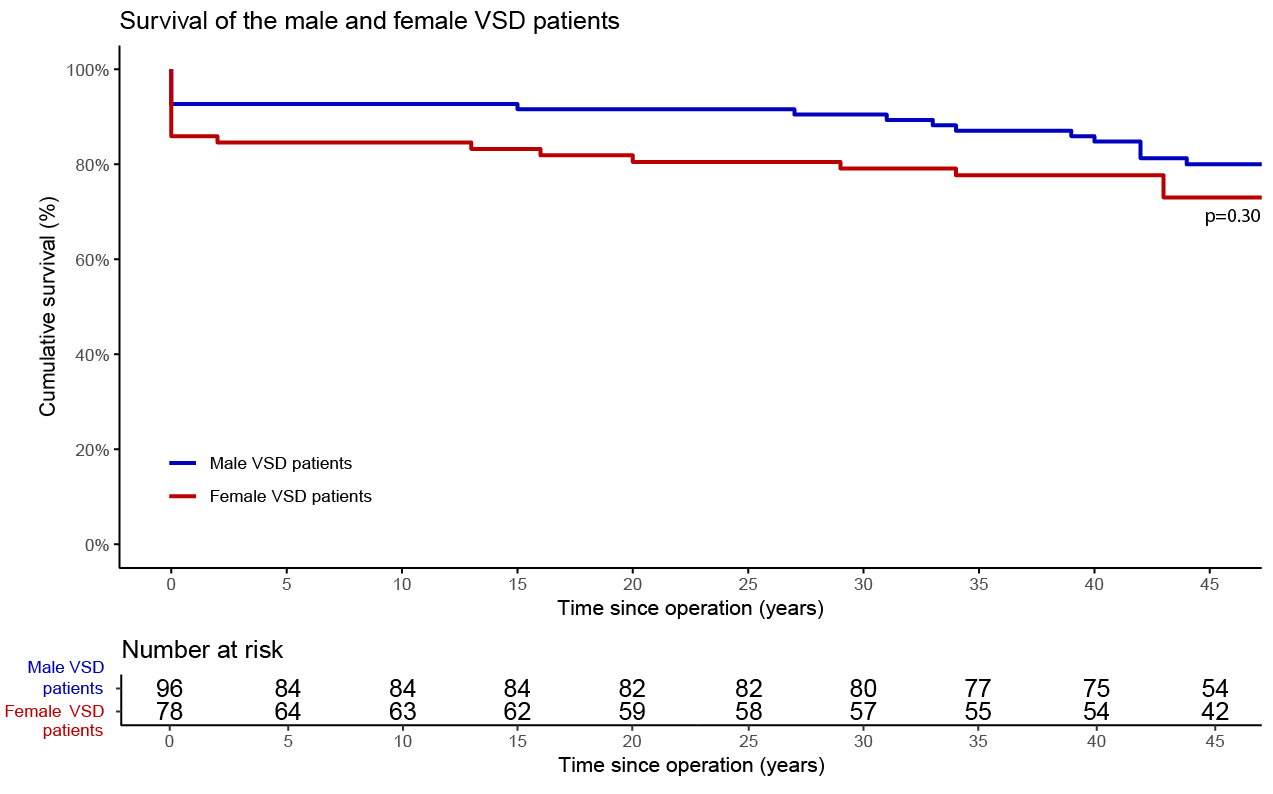  A) Survival plot of the male (at 49 years: 80%, blue) and female VSD patients (at 49 years: 73%, red) |
| --- |
| 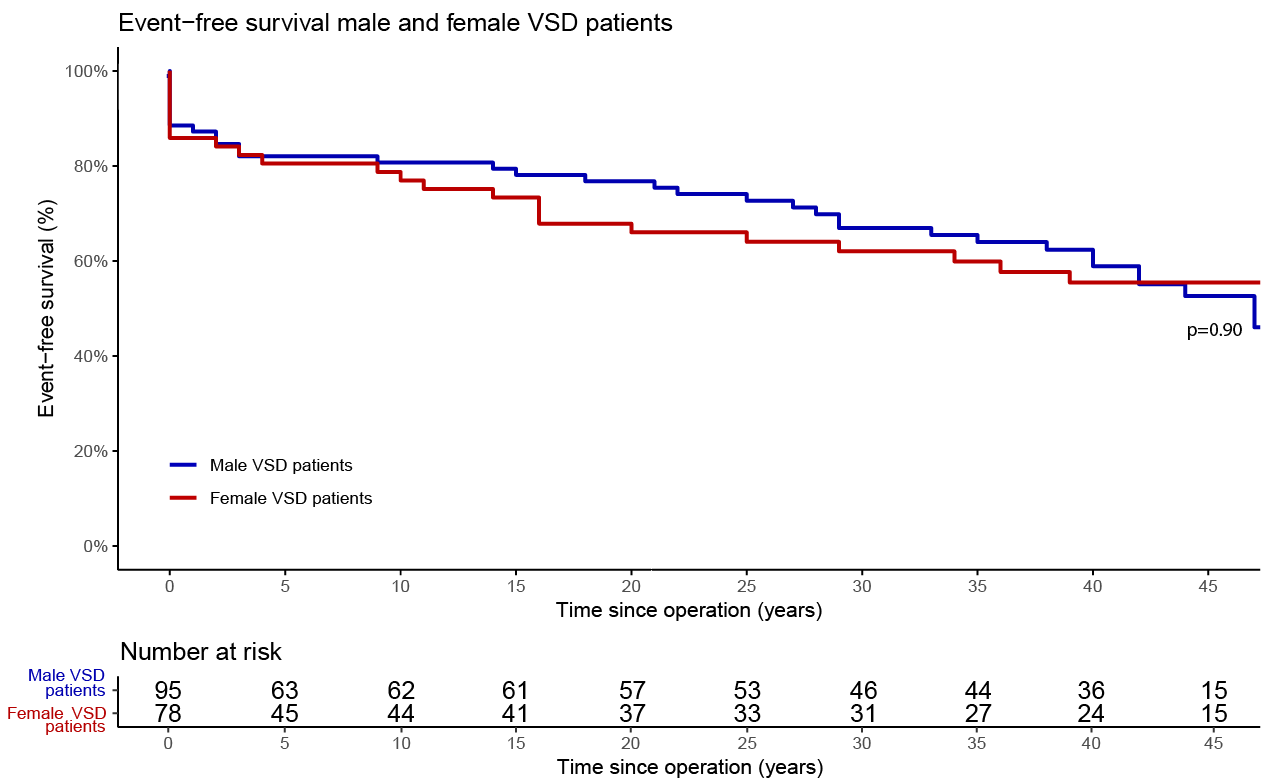  B) Event-free survival of the male (at 49 years: 46%, blue) and female VSD patients (at 49 years: 56%, red) |

**Supplementary Figure 3.** Trend over time diagnostic measurements

|  |  |  |  |  |  |  |  |  |  |  |  |  |
| --- | --- | --- | --- | --- | --- | --- | --- | --- | --- | --- | --- | --- |
| \|  \| \| --- \| |  | p<0.001* |  |  |  |  |  |  |  |  |  |  |
|  |  |  |  |  |  |  |  |  |  |  |  |  |
|  |  |  |  |  |  |  |  |  |  |  |  |  |
|  |  |  |  |  |  |  |  |  |  |  |  |  |
|  |  | p=0.02* |  |  |  |  |  |  |  |  |  |  |
|  |  |  |  |  |  |  |  |  |  |  |  |  |
|  |  |  |  |  |  |  |  |  |  |  |  |  |
|  |  |  | p<0.001* |  |  |  |  |  |  |  |  |  |
|  |  |  |  |  |  |  |  |  |  |  |  |  |
|  |  |  |  |  |  |  |  |  |  |  |  |  |
|  |  |  |  |  |  |  |  |  |  |  |  |  |
|  |  |  |  |  |  |  |  |  |  |  |  |  |
|  |  |  |  |  |  |  |  |  |  |  |  |  |
|  |  |  |  |  |  |  |  |  |  |  |  |  |
|  |  |  |  |  |  |  |  |  |  |  |  |  |
|  |  |  |  |  |  |  |  |  |  |  |  |  |
|  |  |  |  |  |  |  |  |  |  |  |  |  |
|  |  |  |  |  |  |  |  |  |  |  |  |  |
|  |  |  |  |  |  |  |  |  |  |  |  |  |
|  |  |  |  |  |  |  |  |  |  |  |  |  |
|  |  |  |  |  |  |  |  |  |  |  |  |  |
|  |  |  |  |  |  |  |  |  |  |  |  |  |
|  |  |  |  |  |  |  |  |  |  |  |  |  |
|  |  |  |  |  |  |  |  |  |  |  |  |  |

**Supplementary Figure 4A.** 36-Item Short Form Survey results of the VSD cohort compared to 10 years ago

QoL3= 2012; QoL4= 2022;

VSD= ventricular septal defect

p=0.01*

**Supplementary Figure 4B.** 36-Item Short Form Survey results of the VSD cohort over the years

**Supplementary Figure 5.** 36-Item Short Form Survey results of the male and female VSD patients compared to the general Dutch population

GDP= general Dutch population;

VSD= ventricular septal defect

p=0.006*

p=0.009*
