# Supplementary material for: Long-term outcome after surgical ventricular septal defect closure: Longitudinal follow-up into the fifth decade
Source: Int J Cardiol Congenit Heart Dis. 2025 Oct 10;22:100624. doi: 10.1016/j.ijcchd.2025.100624 (PMC12594936; doi:10.1016/j.ijcchd.2025.100624)
Supplement: Multimedia component 2 [file mmc2.docx]

**Supplementary File 1.** *Statistical analysis*

The distributions of continuous variables were tested for normality using the Shapiro-Wilk test and reported as mean ± 1 standard deviation or median [25th-75th percentile], as appropriate. Categorical variables were reported as frequencies and percentages. To examine differences between independent patient groups, the Student's T-test was used for normally distributed continuous variables, the Mann-Whitney U test for non-normally distributed data, and either the χ² test or Fisher’s exact test for categorical variables, depending on expected cell counts. Changes within individuals between visits were also investigated. To evaluate this, the paired T-test was applied for normally distributed continuous variables, the Wilcoxon signed-rank test for skewed data, and the McNemar test for paired categorical outcomes.

For assessing the strength and direction of associations between variables, Pearson’s correlation for normally distributed continuous variables was used and Spearman’s rank correlation for non-parametric data.

To analyze repeated measures and track longitudinal changes within patients across multiple visits, linear mixed-effects models were used for continuous outcomes and generalized linear mixed models for dichotomous outcomes, incorporating random intercepts and slopes to account for multiple measurements per individual. The evaluation time points were included as both fixed and random effects. Random effects were modeled with an unstructured covariance matrix. No additional within-subject correlation structure was specified. For each outcome, two mixed models were constructed: one including both a random intercept and a random slope, and one including only a random intercept. These models were compared using an ANOVA test, and the model with the best fit was selected for each outcome.

Survival of the study cohort was assessed in comparison to the Dutch reference population. Survival curves for the patient cohort were based on individual mortality dates, while survival curves for the general population were derived from publicly available year-specific mortality probabilities. The Dutch reference population was defined as individuals aged 4 years in 1968, corresponding to the median age of primary surgical correction in the cohort. Survival at 49 years was then compared using Z-tests. Additionally, survival analysis was stratified based on whether the VSD was isolated or nonisolated. Cumulative incidence curves for non-fatal outcomes were generated using a nonparametric estimator of cumulative incidence functions.

To identify predictors of long-term outcomes such as mortality and heart failure, univariable Cox proportional hazards regression analyses were performed. The baseline time point was the date of surgery. Variables assessed at the time of operation and included in the models comprised age at operation, era of operation (pre- or post-1975), intraoperative temperature, aorta clamp time per 5 minutes, left atrial saturation, early postoperative arrhythmias, and nonisolated VSD. Variables that showed a statistically significant association with outcomes in the univariable analyses were subsequently included in the multivariable Cox regression model.

Finally, to assess the impact of time-varying variables on survival, Joint Models were used, integrating linear mixed models with Cox regression to evaluate serial measurements in relation to time-to-event outcomes. Time-dependent variables included rhythm on Holter, PR duration, QRS duration, ventricular tachycardia (VT) on Holter, exercise capacity (maximal workload), and LVF and RVF determined via echocardiography.

Statistical analyses were performed using the Statistical Package for Social Sciences (version 28.0, SPSS, Inc, IBM Corp., Chicago, IL, USA) and R software version 4.3.2 (R Foundation for Statistical Computing, Vienna, Austria). All tests were two-sided, and p-values <0.05 were considered statistically significant.
